# Bilateral semilunar perihilar opacities in a patient with pulmonary hypertension

**DOI:** 10.1002/ccr3.1766

**Published:** 2018-08-14

**Authors:** Soulemane Pessinaba, Lantam Sonhaye, Komlavi Yayehd, Latif Amadou, Mohamed Kpelafia, Nsangou Nafissatou, Findibe Damorou

**Affiliations:** ^1^ Cardiology Department Campus Teaching Hospital Lome Togo; ^2^ University of Lome Lome Togo; ^3^ Radiology Department Campus Teaching Hospital Lome Togo

**Keywords:** aneurysm, hypertension, pulmonary artery, thrombosis

## Abstract

Pulmonary arterial aneurysm is a rare entity with a high mortality if left untreated. Pulmonary arterial hypertension is an important cause of PAAs although other infective and auto‐immune causes must be excluded when an aneurysm is identified.

A 50‐year‐old female with a 3‐year medical history of pulmonary arterial hypertension (PAH) was admitted to the hospital for dyspnea, cough, and lower limb edema. Previous evaluations excluded diagnoses such as tuberculosis, Behcet's, syphilis, and other inflammatory diseases in this patient, and PAH was considered idiopathic. The clinical examination revealed blood pressure at 130/80 mm Hg, regular tachycardia at 110 bpm, a loud P2, pulmonary diastolic murmur, and right gallop rhythm. Her chest X‐ray showed cardiomegaly, middle left arch convexity, and bilateral semilunar perihilar opacities (Figure 1).

**Figure 1:**
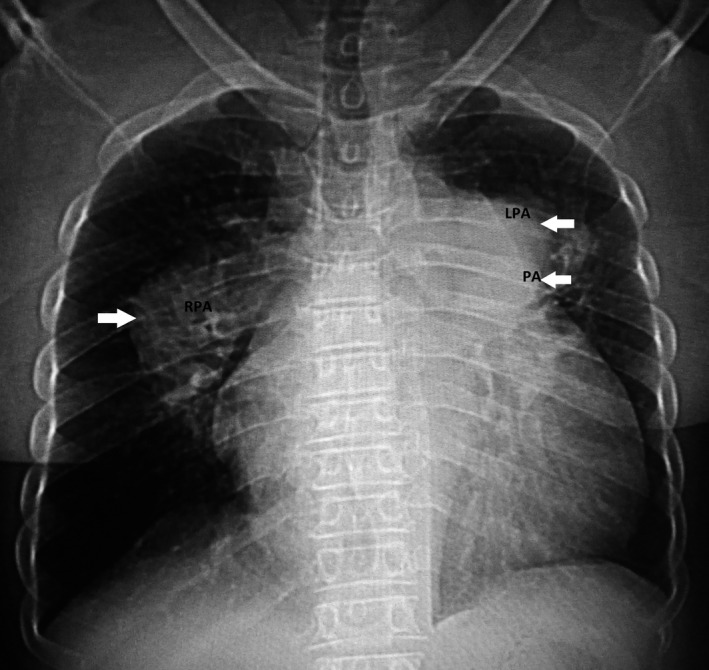
Chest X‐ray showing cardiomegaly and a convexity of the left middle arch, and a bilateral semilunar opacity corresponding to the dilatation of the trunk of pulmonary artery and its branches

Question: What is your diagnosis?

A Pulmonary artery aneurysm?

B Mediastinal tumor?

C Mediastinal adenopathy?

Answer: Pulmonary artery aneurysm. The middle left arch convexity corresponding to the dilatation of pulmonary artery trunk, and the bilateral semilunar opacities corresponding to dilatation of right and left pulmonary arteries.

Transthoracic Doppler echocardiography showed fusiform dilatation of pulmonary artery trunk and its branches with thrombosis ranging from bifurcation to both branches. Systolic pulmonary arterial pressure was 90 mm Hg. The chest CT scan confirmed the aneurysmal dilatation of the pulmonary artery trunk (46 mm) and its branches (42 mm each other) (Figure 2). The patient was placed on heart failure and anticoagulant treatment. The evolution was marked by death in refractory right heart failure.

**Figure 2:**
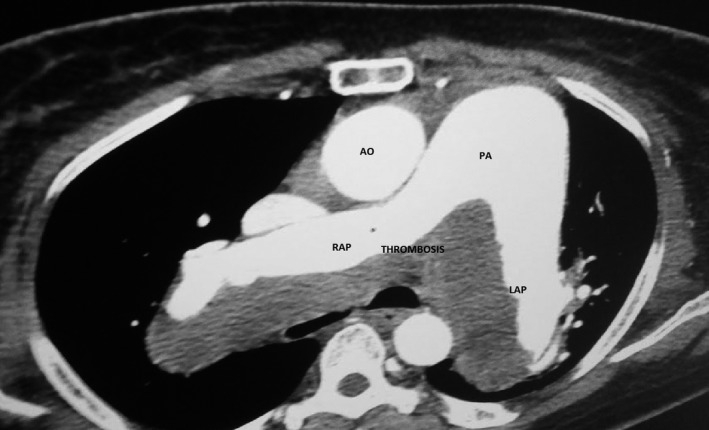
Chest CT scan showing the dilatation of the pulmonary artery trunk (diameter measured at 46 mm) of its right (diameter measured at 42 mm) and left (42 mm diameter) branches and intra‐artery thrombosis

Pulmonary artery aneurysms (PAAs) are rare and infrequently diagnosed. They represent <1% of thoracic aneurysms. Several causes of PAA have been described which can be classified as congenital causes, acquired causes, and idiopathic PAA. Among acquired causes, we have PAH. PAH is an important cause of PAA formation and has been suggested to be a clinical symptom of an existing PAA.^1^ There is no standardized care for PAA. Surgery remains the treatment of choice in the absence of contraindication, to prevent rupture or dissection.^2^

## CONFLICT OF INTEREST

No conflict of interests.

## AUTHORSHIP

SP: wrote and drafted the manuscript, discussed the importance of chest X‐ray initiated in this study, was the operator who made the transthoracic echocardiography. LS and LA: interpreted the chest X‐ray and CT Scan. MK, NN, and KY: did the review of litterature, corrected this article, and made contributions. FD: did the final corrections before the article be submitted. All authors read and approved the final manuscript.
